# COVID-19 Vaccination Engagement and Barriers among Mississippi Pharmacists

**DOI:** 10.3390/pharmacy9040167

**Published:** 2021-10-13

**Authors:** Emily Gravlee, Eric Pittman, Wesley Sparkmon, Hyllore Imeri, Hannah-Faith Cox, Marie Barnard

**Affiliations:** School of Pharmacy, University of Mississippi, University, MS 38677, USA; epittman@olemiss.edu (E.P.); wpsparkm@go.olemiss.edu (W.S.); himeri@go.olemiss.edu (H.I.); hfcox@go.olemiss.edu (H.-F.C.); mbarnard@olemiss.edu (M.B.)

**Keywords:** vaccination, COVID-19, pharmacist, pharmacy, barriers, vaccine hesitancy

## Abstract

After the emergency use authorization of coronavirus disease 2019 (COVID-19) vaccinations in the United States, existing pharmacy infrastructure was leveraged to disseminate vaccines. However, the national uptake of COVID-19 vaccines remains poor. This survey study of Mississippi pharmacists aimed to identify barriers to providing COVID-19 vaccination among pharmacists in practice settings that provided other vaccines. A thematic analysis was used to analyze open-ended survey responses. This study found that the greatest identified barrier to COVID-19 vaccination for pharmacists was patient willingness. The thematic analysis revealed logistical barriers, vaccine hesitancy, and rural pharmacy distribution concerns. These findings suggest that pharmacists require further training in overcoming vaccine hesitancy, and potentially indicate a need for the distribution of vaccination responsibilities to additional pharmacy staff members.

## 1. Introduction

In December 2020, the United States (US) Food and Drug Administration (FDA) issued emergency use authorizations for both the Pfizer-BioNTech and Moderna coronavirus disease 2019 (COVID-19) vaccines, and, two months later, authorized the Janssen COVID-19 vaccine for emergency use [1]. By July 2021, almost half of the adult US population had fully been vaccinated, with 56% of adults receiving at least one COVID-19 vaccination [2]. A crucial driver for COVID-19 vaccinations in the US has been the Federal Retail Pharmacy Program for COVID-19 Vaccination. This program authorizes certain retail pharmacies, called pharmacy partners, to receive a COVID-19 vaccine supply directly from the federal government, and has been implemented in over 40,000 retail pharmacies across the United States [3]. Since the program launched in February 2021, approximately 99 million COVID-19 vaccinations have been administered in retail pharmacy settings, highlighting the pharmacists’ role as a key factor in the federal government’s COVID-19 vaccination strategy [3]. 

Despite both public and private engagement in COVID-19 US vaccination efforts, there is a suboptimal uptake of COVID-19 vaccine in the States. While over half of US adults report receiving at least one vaccine, uptake lags sorely behind the national goal of 80% [2], and the average number of daily vaccinations has declined by approximately 85% since April 2021 [4]. The Kaiser Family Foundation reported that the percentage of individuals who had received one dose of vaccine or intended to do so increased from 61% to 64% from March to April 2021, or by only three percentage points [5]. Given pharmacy accessibility and existing federal resource allocation, improved pharmacy-based vaccination services may increase COVID-19 vaccine uptake. To improve existing services, a greater understanding of barriers to engagement with vaccination efforts among pharmacies is needed. Understanding barriers to pharmacists offering COVID-19 vaccination can inform pointed interventions to improve vaccine provision in the pharmacy setting. Additionally, the characterization of pharmacies providing COVID-19 vaccine services may highlight a potential disparity in how COVID-19 vaccines are administered, and may identify subsequent opportunity for intervention. Specifically, pharmacies may differ regarding the access, rurality, staffing, pharmacy type, and utilization of pharmacy technicians in COVID-19 vaccinations. Together, these aims may facilitate a greater understanding of tailored interventions that could improve vaccination numbers. 

While a national issue, concerns around the low vaccine uptake in the South are especially pertinent as health behaviors and sociodemographic factors may place these individuals at a greater risk for severe COVID-19 disease [6,7]. Additionally, rurality and fragmented access to vaccines may impact COVID-19 vaccine uptake. Notably, Mississippi has the second-lowest rate of COVID-19 vaccinations in the country [4]. While 47.2% of adults have received at least one vaccine dose, only one-third of the total population is fully vaccinated [8]. Gaps in COVID-19 vaccination status and the opportunity for pharmacist involvement highlights the need to explore Mississippi pharmacists’ engagement in COVID-19 vaccine administration and barriers to vaccination during the pandemic. This study aims to describe the engagement of pharmacists in COVID-19 vaccine administration in Mississippi, identify barriers to pharmacist engagement in COVID-19 vaccinations across Mississippi, and assess the influence of pharmacist and practice site factors on vaccination initiation in practice settings where vaccinations are provided across Mississippi. 

## 2. Materials and Methods

### 2.1. Study Design and Sample

A cross-sectional survey was conducted among Mississippi pharmacists using Qualtrics, an online survey software system. Questions were conceptualized by the author team to meet the study objectives. The full survey is available in Supplementary Materials File S1 . The survey was distributed via email to pharmacists through four Mississippi professional pharmacy organizations: Mississippi Pharmacists Association, Magnolia State Pharmaceutical Society, Mississippi Independent Pharmacy Association, and Mississippi Society of Health-System Pharmacists. Pharmacists whose emails were on-file with each professional organization received email invitations to complete the survey. The survey was distributed to approximately 3500 pharmacists beginning in April 2021 and was available for completion for approximately four weeks. After initial dissemination, no reminders to complete the survey were sent. Respondents who completed the survey were given the option to be entered into a raffle to receive one of two USD 200 gift cards as incentives to complete the survey. The study was conducted according to the guidelines of the Declaration of Helsinki and approved by the Institutional Review Board of University of Mississippi (protocol code 9x-003-1 and approved 22 April 2021).

### 2.2. Data Analysis

Descriptive statistics were calculated to characterize the sample, vaccinations provided, and perceived barriers to COVID-19 vaccinations. A Chi-square test was used to determine if differences in vaccination initiation engagement existed between PharmD and RPh pharmacists. Quantitative data were analyzed with SPSS v26. Qualitative data were analyzed by thematic analysis. Two authors coded open-ended responses to the prompt, “Please share any other comments about COVID-19 vaccinations in the pharmacy practice setting.”

## 3. Results

### 3.1. Participant Characteristics

Of approximately 3500 distributed surveys, 273 responses were received. Of these, 233 provided responses to the item, “Does your pharmacy setting provide any vaccination administration services?” These 233 responses comprised the analytic data set, resulting in an estimated response rate of 6.66%. Descriptive characteristics of responding pharmacists can be found in Table 1. Almost half of responding pharmacists were less than forty-one years old (*n* = 111, 47.7%) and just over half were female (*n* = 130, 55.8%). Most surveyed pharmacists indicated that they practiced in a community pharmacy setting (*n* = 157, 67.4%). Sixty-two percent (*n* = 145) of responding pharmacists indicated that they were trained as Doctors of Pharmacy (PharmDs), and 35.6% (*n* = 83) indicated that they were trained as registered pharmacists (RPh). Of 233 total responses, 179 pharmacists responded to the question regarding weekly prescription volume. Weekly prescription volume varied, with a small percentage (10.6%) filling more than 4001 prescriptions per week in their pharmacy.

### 3.2. Pharmacist Engagement in Vaccine Administration

**Figure 1:**
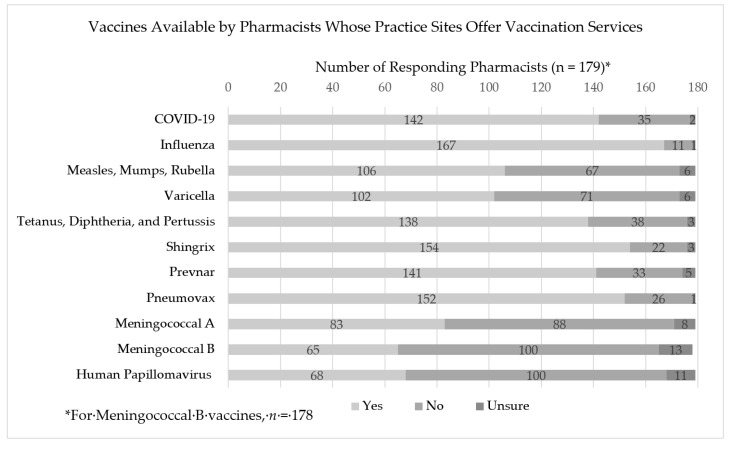
Vaccines available by pharmacists whose practice sites offer vaccination services.

Overall, 79.0% (*n =* 184) of 233 responding pharmacists indicated that their practice setting provides vaccination administration services. Approximately 17% of responding pharmacists (*n =* 39) indicated that their practice site did not provide vaccination services, and 4.3% (*n =* 10) of responding pharmacists were unsure if their practice site provided vaccination services. Of pharmacists responding that their practice settings are involved in vaccination administration, 79.3% (*n =* 142) indicated that they offer COVID-19 vaccines at their practice site. Interestingly, COVID-19 vaccines were the fourth most commonly available vaccine for administration in these practice settings, lagging only behind influenza (93.3%, *n =* 167), Shingrix (86.0%, n *=* 154), and Pneumovax (84.9%, *n =* 152) vaccinations. Vaccines available where pharmacists’ practice sites offer vaccination services are described in Figure 1. 

Pharmacists were also asked about their engagement in the initiation of vaccination in their patient population. Most indicated that they ask patients at least some of the time if they are interested in some vaccinations. There was no significant difference in vaccination initiation between respondents who had a PharmD compared to those with an RPh, *X*^2^(4, *N* = 174) = 5.52, *p =* 0.238 (Table 2).

### 3.3. Barriers to Pharmacist Vaccine Administration

Pharmacists were asked to indicate how significant certain factors are as barriers to providing COVID-19 vaccination services. Each factor was rated on a scale of 0 (not a barrier) to 10 (a significant barrier). The strongest barriers to COVID-19 vaccination administration were patient willingness, time limitations, and staffing. Practice regulations, pharmacists’ lack of knowledge around COVID-19 vaccinations, and personnel resistance were the least challenging barriers to overcome. Descriptive statistics for barriers to provision of COVID-19 vaccinations can be found in Table 3.

### 3.4. COVID-19 Vaccination Services among Mississippi Pharmacists

Forty-nine pharmacists responded to the open-ended prompt, “Please share any other comments about COVID-19 vaccinations in the pharmacy practice setting.” Qualitative thematic analysis of responses to the prompt revealed three main themes: logistical barriers, vaccine hesitancy, and slow distribution to independent pharmacies. Within the theme of vaccine barriers, sub-themes of staffing problems, fear of vaccine waste, and the recording or regulatory requirements emerged. Representative participant quotes are present in Table 4.

#### 3.4.1. Logistical Barriers

Responses indicated that Mississippi pharmacists feel overwhelmed by the burden of administering vaccines in addition to the usual work flow, and, in particular, the lack of personnel to administer vaccinations challenges and frustrates pharmacy staff. One pharmacist noted, “COVID(-19) vaccinations were add[ed] to the daily work without additional personnel or scheduling more hours”, while another noted that, “Vaccines are yet another add on service that is not taken into account when determining staffing issues. …this only adds to the stress of a normal day.” Fear of vaccine waste was also a common comment by pharmacists. Balancing supply and demand for the COVID-19 vaccine for individual pharmacies is especially challenging in the face of vaccination expiration and patient vaccination hesitancy. One pharmacist noted that, “…it has become very overwhelming with making sure we do not waste doses while also having vaccine available for patients who want to get it…”, while another noted that, “It is becoming increasingly difficult to not waste vaccine and the main factor seems to be lack of interest from patients.”

Finally, pharmacists noted that reporting or regulatory requirements for COVID-19 vaccination acted as barriers to vaccine administration. Pharmacists indicated that they were delayed in their ability to provide vaccines due to long training hours for vaccination certification. One pharmacist noted that, “…I feel fully capable to administer vaccines but I am not allowed until I finish…[the] training program. This has been an obstacle for me to give vaccines.” Additionally, reporting requirements were burdensome to some pharmacy staff and prevented optimum workflow. Where vaccination training may have felt unnecessary to pharmacists, some pharmacists noted that the lack of training with reporting systems hampered their ability to provide efficient care. One individual noted that, “It has been difficult scheduling COVID vaccines, completing paper work, and completing all other activities…and submitting shot records within MIIX [Mississippi Immunization Information eXchange] was tough with no initial training.”

#### 3.4.2. Vaccine Hesitancy

Vaccine hesitancy emerged as a second theme from the open-ended response prompt. Pharmacists noted that many COVID-19 vaccines are not administered simply because patients are not willing to receive them, and that many patients endorse vaccine misinformation. One pharmacist claimed that, “Many patients [are] worried about fertility”, while another noted the “continuous decrease in numbers...” of patients willing to receive the vaccine due to “uncertainty…by patients”. Another pharmacist tied in the lack of patient interest in COVID-19 vaccination to waste, claiming that “it’s difficult to get 10 people to commit for one day to Moderna [vaccination]…many do not show up for their appointment, leaving us in a rush to find someone so that there isn’t waste.” Additionally, while patients may receive their first vaccine, patients may not show up for their second scheduled dose, with one pharmacist noting that it is, “sometimes challenging to get people to return for their second injection.” Overall, pharmacists indicated that patient hesitancy is a significant challenge to the administration and stocking of the COVID-19 vaccine.

#### 3.4.3. Slow Distribution to Independent Pharmacies

An additional theme that emerged from the open-ended response prompt was the slow distribution of the COVID-19 vaccines to community pharmacies. Pharmacists noted that shipment delays seemed to be due to delays with contracted distributors, and that these delays resulted in missed opportunities to provide care. One pharmacist claimed, “We planned on administering the COVID(-19) vaccine but had the hardest trouble to get [the stock distributor] to send us our shipment. Now I feel like everyone that was waiting to get it here has gotten it somewhere else.” Pharmacists noted that the delay to stock the COVID-19 vaccination in independent pharmacies, especially in rural settings, where independent pharmacies may be the only potential vaccination providers, may have impeded vaccine administration and put rural populations at risk. One pharmacist stated, “In a state like Mississippi with many rural communities many people did not have access to the vaccine early because there were not sites locally…Independent community pharmacies could have accelerated the process of vaccine connections in rural communities.” Overall, independent pharmacists noted frustration with the poor or slow distribution to their pharmacies. One pharmacist questioned, “Why were independent pharmacies left out?”

## 4. Discussion

This study found that most Mississippi pharmacy practice locations offering vaccination services provided COVID-19 vaccinations. Additionally, despite being very new to the market, COVID-19 vaccines were the fourth most commonly offered vaccines in practice locations that did offer vaccination services in Mississippi. Despite COVID-19 vaccines being available, however, patient uptake in Mississippi remains low. As production and affordability barriers in the US are minimal [9], the difficulty in ensuring vaccine accessibility, adequate supply, and public confidence may be large remaining barriers to uptake [10].

The pharmacist responses to this survey study suggest that the uptake of COVID-19 vaccination services by pharmacists in Mississippi is limited by both logistical and patient barriers. This study found that the greatest identified barrier to COVID-19 vaccination among pharmacists practicing at settings that offered vaccine services was patient willingness. The thematic analysis of comments regarding COVID-19 vaccination corroborates vaccine hesitancy, defined as a delay in acceptance or refusal of vaccination despite available services [11], as a principal issue in COVID-19 uptake in Mississippi. Pharmacists suggested that patients do not wish to be vaccinated, or that they endorse vaccine misinformation. This finding is likely not unique to Mississippi. A lack of public confidence in the COVID-19 vaccine in the US is a chief concern. In fact, up to one-third of US adults do not plan to be vaccinated [12]. Among individuals who refuse the vaccine, the Kaiser Family Foundation found that 53% of unvaccinated surveyed adults indicated that vaccine newness or concern over side effects are major barriers to uptake [13]. The same survey found that 38% of respondents do not believe that they need the vaccine [13]. While discouraging, it should be noted that vaccine hesitancy is fluid, and that only 11% of the Kaiser Family Foundation survey respondents indicated that their main reason for vaccine delay was vaccine refusal [13].

Pharmacist involvement in overcoming vaccine hesitancy may be critical in encouraging the public uptake of COVID-19 vaccines. The 5C model of vaccination hesitancy notes that psychological drivers of vaccine uptake behavior include confidence, complacency, convenience, risk calculation, and collective responsibility [14,15]. Pharmacists have the training and opportunity to work with patients to build confidence, reduce the patient-perceived vaccine risk, and promote collective responsibility for COVID-19 vaccination [16]. Professional pharmacy organizations have recognized the important role that pharmacists can play in promoting COVID-19 vaccine uptake and have released training resources for pharmacists to overcome COVID-19 vaccine hesitancy. The American Pharmacists Association (APhA) created the Vaccine Confident website (available online: www.vaccineconfident.pharmacist.com; accessed on 29 July 2021), which provides information on promoting vaccine uptake among a variety of communities and provides opportunity for continuing education credit on various topics surrounding vaccine hesitancy, including motivational interviewing and strategies for recommending vaccines [17]. The American Society of Health Systems Pharmacists (ASHP) has also released continuing education on addressing COVID-19 vaccine hesitancy [18]. While overcoming vaccine hesitancy may seem daunting or time-consuming to many pharmacy practitioners, pharmacists are in a unique position to provide helpful information, reach out to communities, and overcome hesitancy. Additionally, training and resources are available to assist in this important task. Pharmacists should be informed on how to overcome vaccine hesitancy, not only for the health of their practices, but for the health of their communities.

The majority of pharmacy practitioners, regardless of PharmD or RPh training, indicated that they regularly review charts for immunization indications. Despite pharmacists reporting activity identifying indicated vaccines, pharmacists in this study described logistical barriers, such as time limitations, poor staffing, and scheduling conflicts, that served as barriers to COVID-19 vaccine dissemination. This may indicate a need for other pharmacy staff members, such as pharmacy technicians, to either participate in vaccination delivery or chart review for vaccine indications. Notably, the Pharmacy Technician Certification Board and the American Pharmacists Association worked together to develop a pharmacy technician vaccination certification program, which launched in February 2021 [19]. While some states have allowed pharmacy technicians to perform vaccination-related duties, such as administration, vaccine history assessment, record completion, and billing, prior to the program’s release [20], more states, including Mississippi [21], have authorized technician-administered vaccinations amid the COVID-19 pandemic. 

Open-ended responses to the survey revealed that community pharmacists in rural settings feel that they have been left out of COVID-19 vaccine dissemination. This finding is consistent with a policy brief released by the Rural Policy Research Institute (RUPRI), which found that up to 442 of 1962 non-metropolitan counties in the United States lacked a pharmacy eligible for pharmacy partner status under the Federal Retail Pharmacy Program for COVID-19 Vaccination [22]. While the Federal Retail Pharmacy Program for COVID-19 Vaccination allowed broad access to COVID-19 vaccinations across the United States, it may have represented a misstep for rural wellness. A lack of attention to gaps in care among rural spaces potentially perpetuated the poor vaccine uptake among these communities, which are typically older, poorer, and sicker than urban counterparts [23]. While pharmacists may influence COVID-19 vaccination uptake by leveraging pharmacy technician partners and providing education to overcome vaccine hesitancy, these efforts may be diminished by a lack of convenient or feasible spaces for rural patients to receive vaccinations. This missed opportunity for rural health should inform future broad vaccine dissemination interventions, and highlights the potential need for mobile vaccination clinics across states with pockets of hard-to-reach populations [24].

The limitations of the study include a low overall response rate and potential response bias. Selection bias may exist due to the nature of the survey. This survey was only available in English and was distributed via email lists of professional pharmacy organizations. The survey may exclude pharmacists with a limited English proficiency, poor internet literacy, or those who are not affiliated with state professional pharmacy organizations. Additionally, because this was an anonymous survey distributed at a pharmacist-level, there is no way to know if duplicate pharmacies are represented within the collected data or if collected data are geographically representative of the state. However, this study provides unique insight into pharmacist vaccination engagement and barriers in Mississippi during the COVID-19 pandemic. While the study was performed among a small sample of Mississippi pharmacists, the authors believe that findings on barriers to vaccination and thematic findings are broadly applicable across the United States. The authors suggest further studies on barriers of COVID-19 vaccination within community pharmacy settings, and assessments of pharmacy technician vaccination engagement, barriers to COVID-19 vaccination, and pharmacists’ perceptions of pharmacy technician involvement in COVID-19 vaccination. 

## 5. Conclusions

This study found that pharmacists’ greatest perceived barrier to providing coronavirus disease 2019 (COVID-19) vaccinations in Mississippi is patient willingness. Additional training on vaccine hesitancy provided by professional organizations may assist pharmacists in encouraging the uptake of COVID-19 vaccinations to improve vaccination rates and public protection against COVID-19. Considering the growing problem of vaccine hesitancy, as well as its role in the COVID-19 pandemic, an increased pharmacy curriculum emphasis on overcoming vaccine hesitancy may better prepare future pharmacists to address vaccine hesitancy issues and promote public health. Leveraging pharmacy technician partners in vaccination administration, documentation, and billing may alleviate logistical barriers related to COVID-19 vaccine administration, and may allow for an increased COVID-19 vaccination uptake. Finally, while federal programs have allowed broad access for COVID-19 vaccinations, the slow COVID-19 vaccine dissemination to rural spaces may have been a missed opportunity for rural health.

## Figures and Tables

**Table 1:** Descriptive characteristics of responding pharmacists.

Characteristic	Response	*n*	%
Age *	Less than 30	40	17.2
	30–40	71	30.5
	41–50	42	18.0
	51 and Above	80	34.3
Gender *	Male	99	42.5
	Female	130	55.8
	Other/Prefer Not to Report	4	1.7
Training Completed *	RPh	83	35.6
	PharmD	145	62.2
	Residency Training	33	14.2
	PhD	3	1.3
	Other (MBA, MPH, etc.)	16	6.9
Years Actively Practicing *	0–3	35	15.0
	4–10	60	25.8
	11–20	44	18.9
	21 or More	94	40.3
Primary Practice Setting *	Hospital/Institutional Pharmacy	38	16.3
	Community Pharmacy	157	67.4
	Specialty Pharmacy	7	3.0
	Other	31	13.3
Primary Practice Location *	Rural	109	46.8
	Urban	69	29.6
	Suburban	55	23.6
Average Prescriptions per Week ^†^	Under 1000	32	17.8
	1000–2000	59	33.0
	2001–4000	69	38.5
	4001 and Above	19	10.6

* Percentages calculated from *n* = 233 responding pharmacists. ^†^ Percentages calculated from *n* = 179 responding pharmacists.

**Table 2:** Pharmacists’ engagement in vaccination initiation.

To what Extent Are You Involved in Initiating Immunization …	PharmD % (*n*)	RPh % (*n*)
Not at all—I do not ask about nor recommend immunizations	5.9% (7)	10.7% (6)
I follow the requirements of my practice setting but in general I do not believe pharmacists should spend time on unsolicited immunization education	5.1% (6)	5.4% (3)
I will ask patients if they are interested in receiving some immunizations (e.g., influenza) but I do not regularly ask about or recommend immunizations	48.3% (57)	33.9% (19)
I regularly review patient records and recommend immunizations for which they are eligible even if that is not the reason they have come to the pharmacy	35.6% (42)	48.2% (27)
Other	5.1% (6)	1.8% (1)

**Table 3:** Perceived barriers to COVID-19 vaccination among surveyed pharmacists.

Barrier	*N*	Mean	SD
Vaccine Availability	204	4.3	3.8
Vaccine Storage	204	4.6	3.6
Space Availability	204	3.7	3.3
Staffing	203	6.2	3.6
Lack of Knowledge	203	3.3	2.9
Scheduling	202	5.6	3.4
Time Limitations	205	6.4	3.6
Patient Willingness	202	6.5	3.1
Practice Regulations	202	3.1	2.5
Reimbursement	203	4.4	3.3
Personnel Resistance	203	3.3	2.9

**Table 4:** Representative thematic quotes.

Theme	Sub-Themes	Representative Participant Quotes
Logistical Barriers	Staffing Problems	“This was an overwhelming burden to put on pharmacy …. Most companies added little to no extra help for this additional load of work.”
	Fear of Vaccine Waste	“Package size and availability is the primary concern at our pharmacy …. We currently do not give COVID vaccinations due to multi-use vials that must be completed within a few hours of breaking the seal.”
	Recording or Regulatory Requirements	“It has been difficult scheduling COVID vaccines, completing paper work and completing all other activities. Administering COVID vaccines is very time consuming and submitting shot records within MIIX [Mississippi Immunization Information eXchange] was tough with no initial training.”
Vaccine Hesitancy	--	“[There is a] Continuous decrease in [patient] numbers [to receive the vaccine]. Uncertainty on [the COVID-19] shot by patients is huge. [There are] Negative feelings from customers on the vaccine being a government scam …”
Slow Distribution to Community Pharmacies	--	“We planned on administering the Covid vaccine but have had the hardest trouble to get [the stock distributor] to send us our shipment. Now I feel like everyone that was waiting to get it here has gotten it from somewhere else.”

## Data Availability

The datasets generated during and/or analyzed during the current study are available from the corresponding author on reasonable request.

## Supplementary Materials

The following are available online at https://www.mdpi.com/article/10.3390/pharmacy9040167/s1, File S1: Immunization Survey 31 March 2021.
