# Supplementary material for: COVID-19 Vaccination Engagement and Barriers among Mississippi Pharmacists
Source: Pharmacy (Basel). 2021 Oct 13;9(4):167. doi: 10.3390/pharmacy9040167 (PMC8544717; doi:10.3390/pharmacy9040167)
Supplement: Supplementary file 1 [file pharmacy-09-00167-s001.zip › pharmacy-1385669-supplementary.pdf]

Default Question Block

YOUR INPUT IS IMPORTANT!

Thank you for helping us understand immunizations in the pharmacy practice setting. Your feedback may help public and private payers determine how to establish policy and reimbursement for immunization services in pharmacies.

Description: The purpose of this research project is to understand the current engagement in immunization services in pharmacy practice settings in Mississippi and to explore pharmacists' potential interest in providing these services. You will not be asked for your name or any other identifying information.
Cost and Payments: It will take you approximately five minutes to complete this survey. You will have the option at the end of the survey to be *entered into a raffle for one of two $200 Amazon gift cards.*
Risks and Benefits: We do not think there are any risks associated with completing this survey. Your responses may help develop immunization administration and reimbursement policies.
Confidentiality: No identifiable information will be recorded, therefore we do not think you can be identified from this study.
Right to Withdraw: You do not have to take part in this study and you may stop participation at any time. If you start the study and decide that you do not want to finish, all you have to do is to exit the survey.
IRB Approval: This study has been reviewed by The University of Mississippi's Institutional Review Board (IRB). If you have any questions, concerns, or reports regarding your rights as a participant of research, please contact the IRB at (662) 915-7482 or irb@olemiss.edu.

I have read and understand the above information. By completing the survey I consent to participate in the study.

PLEASE NOTE! This survey is being shared through multiple state associations. Please answer the survey only one time.

DIRECTIONS: Your responses are completely anonymous. No one will be able to associate your responses with you. Please complete each item to the best of your ability. If you are uncertain of an exact response, please estimate to the best of your ability.

What is your current age in years?

- ◯ I am under 18 years of age
- ◯ Less than 30
- ◯ 30-40
- ◯ 41-50
- ◯ 51 and above

Please indicate your gender:

- ◯ Male

- ◯ Female
- ◯ Other
- ◯ Prefer Not to Report

Which best describes your most pharmacy training? *Check all that apply.*

- ☐ RPh
- ☐ PharmD
- ☐ Completed Residency Training
- ☐ PhD
- ☐ Other (e.g., MPH, MBA, etc.): ____________

For how many years have you been actively practicing pharmacy?

- ◯ 0-3 years
- ◯ 4-10 years
- ◯ 11-20 years
- ◯ 21 or more years

Which of the following best describes your primary practice setting?

- ◯ Hospital/Institutional Pharmacy
- ◯ Community Pharmacy
- ◯ Specialty Pharmacy
- ◯ Other - please describe: ____________

Which of the following best describes the setting of your primary practice setting?

- ◯ Rural
- ◯ Urban
- ◯ Suburban

On average, how many prescriptions per week does your pharmacy fill? (new and refills)

- ◯ Under 1,000
- ◯ 1,000-2,000
- ◯ 2,001-4,000
- ◯ 4,001 and above
- ◯ Uncertain
- ◯ Not applicable

To what extent are you involved in key decision-making related to implementation of new products or services for your pharmacy?

- ◯ Not at all
- ◯ A little
- ◯ Some
- ◯ A good amount
- ◯ To a great extent

Have you completed any certifications or training in immunization administration?

- ◯ Yes
- ◯ No
- ◯ Unsure

Does your practice setting provide any immunization administration services?

- ◯ Yes
- ◯ No
- ◯ Unsure

Were you certified in immunization administration **BEFORE** COVID-19 vaccinations were available?

- ◯ Yes
- ◯ No
- ◯ Unsure

Did your practice setting provide any immunization administration services **BEFORE** COVID-19 vaccinations were available?

- ◯ Yes
- ◯ No
- ◯ Unsure

To what extent are you involved in initiating immunization?

- ◯ Not at all- I do not ask about nor recommend immunizations
- ◯ I follow the requirements of my practice setting but in general I do not believe pharmacists should spend time on unsolicited immunization education
- ◯ I will ask patients if they are interested in receiving some immunizations (e.g., influenza) but I do not regularly ask about or recommend immunizations
- ◯ I regularly review patient records and recommend immunizations for which they are eligible even if that is not the reason they have come to the pharmacy
- ◯ Other (please describe): ____________

Do you administer the following routine immunizations in your practice setting?

	Yes	No	Unsure
COVID-19	◯	◯	◯
Influenza	◯	◯	◯
Measles, Mumps, and Rubella	◯	◯	◯
Varicella	◯	◯	◯
Tetanus, Diphtheria, and Pertussis	◯	◯	◯
Zostavax	◯	◯	◯
Shingrix	◯	◯	◯
Prevnar	◯	◯	◯
Pneumovax	◯	◯	◯
Menactra or Menveo (Meningococcal A)	◯	◯	◯
Bexsero or Trumenba (Meningococcal B)	◯	◯	◯
Gardasil or Cervarix (HPV)	◯	◯	◯

When your pharmacy provides an immunization, which costs are reimbursed by each of the following payers (check all that apply):

	Drug Cost	Administration Fee	Dispensing Fee	I don't know
Medicaid	☐	☐	☐	☐
Medicare	☐	☐	☐	☐
Commercial Insurance	☐	☐	☐	☐

How many COVID-19 immunizations does your practice setting administer in an average week?

- ◯ 0-10
- ◯ 11-20
- ◯ 21-50
- ◯ 51+
- ◯ Uncertain

During flu season, how many influenza immunizations does your practice setting administer in an average week?

- ◯ 0-10
- ◯ 11-20
- ◯ 21-50
- ◯ 51 +
- ◯ Uncertain

Excluding influenza immunizations, how many immunizations does your practice setting administer in an average week?

- 0-1
- 2-5
- 6-7
- 8-10
- Greater than 10
- Unsure

Excluding COVID-19 immunizations, how many immunizations does your practice setting administer in an average week?

- 0-1
- 2-5
- 6-7
- 8-10
- Greater than 10
- Unsure

Excluding COVID-19 and influenza immunizations, how many immunizations does your practice setting administer in an average week?

- 0-1
- 2-5
- 6-7
- 8-10
- Greater than 10
- Unsure

How many immunizations does your practice setting administer in an average week?

- 0-1
- 2-5
- 6-7
- 8-10
- Greater than 10
- Unsure

Are you interested in administering the following immunizations that you do not currently provide in your practice setting?

	Not interested	Potentially interested	Definitely interested
» COVID-19	○	○	○
» Influenza	○	○	○
» Measles, Mumps, and Rubella	○	○	○

	Not interested	Potentially interested	Definitely interested
» Varicella	◯	◯	◯
» Tetanus, Diphtheria, and Pertussis	◯	◯	◯
» Zostavax	◯	◯	◯
» Shingrix	◯	◯	◯
» Prevnar	◯	◯	◯
» Pneumovax	◯	◯	◯
» Menactra or Menveo (Meningococcal A)	◯	◯	◯
» Bexsero or Trumenba (Meningococcal B)	◯	◯	◯
» Gardasil or Cervarix (HPV)	◯	◯	◯

How interested are you in administering the following vaccinations in your practice setting?

	Not interested	Potentially interested	Definitely interested
COVID-19	◯	◯	◯
Influenza	◯	◯	◯
Measles, Mumps, and Rubella	◯	◯	◯
Varicella	◯	◯	◯
Tetanus, Diphtheria, and Pertussis	◯	◯	◯
Zostavax	◯	◯	◯
Shringrix	◯	◯	◯
Prevnar	◯	◯	◯
Pneumovax	◯	◯	◯
Menactra or Menveo (Meningococcal A)	◯	◯	◯
Bexsero or Trumenba (Meningococcal B)	◯	◯	◯
Gardasil or Cervarix (HPV)	◯	◯	◯

Do you administer the following "catch-up" immunizations in your practice setting?

	Yes	No	Uncertain
Hepatitis B	◯	◯	◯
Hepatitis A	◯	◯	◯
Rotavirus	◯	◯	◯
DTaP	◯	◯	◯
Hib	◯	◯	◯
IPV	◯	◯	◯

How interested are you in administering the following "catch up" immunizations that you do not currently provide in your practice setting?

	Not interested	Potentially interested	Definitely interested
» Hepatitis B	◯	◯	◯
» Hepatitis A	◯	◯	◯

	Not interested	Potentially interested	Definitely interested
» Rotavirus	◯	◯	◯
» DTaP	◯	◯	◯
» Hib	◯	◯	◯
» IPV	◯	◯	◯

How interested are in administering the following "catch up" immunizations that you do not currently provide in your practice setting?

	Not Interested	Potentially Interested	Definitely Interested
Hepatitis B	◯	◯	◯
Hepatitis A	◯	◯	◯
Rotavirus	◯	◯	◯
DTaP	◯	◯	◯
Hib	◯	◯	◯
IPV	◯	◯	◯

For all immunizations that you do not currently offer at your practice setting, what costs would have to be reimbursed for you to consider offering these immunizations? *Check all that apply.*

- ☐ Drug Costs
- ☐ Administration Fee
- ☐ Dispensing Fee

Please indicate how significant the following factors may be as barriers **to providing COVID-19 immunization services** in your practice setting.

	0 = not a barrier	1	2	3	4	5	6	7	8	9	10 = significant barrier
Vaccine Availability	◯	◯	◯	◯	◯	◯	◯	◯	◯	◯	◯
Vaccine Storage	◯	◯	◯	◯	◯	◯	◯	◯	◯	◯	◯
Space Availability	◯	◯	◯	◯	◯	◯	◯	◯	◯	◯	◯
Staffing	◯	◯	◯	◯	◯	◯	◯	◯	◯	◯	◯
Lack of Knowledge	◯	◯	◯	◯	◯	◯	◯	◯	◯	◯	◯
Scheduling	◯	◯	◯	◯	◯	◯	◯	◯	◯	◯	◯
Time limitations	◯	◯	◯	◯	◯	◯	◯	◯	◯	◯	◯
Patient Willingness	◯	◯	◯	◯	◯	◯	◯	◯	◯	◯	◯
Practice Regulations	◯	◯	◯	◯	◯	◯	◯	◯	◯	◯	◯
Reimbursement	◯	◯	◯	◯	◯	◯	◯	◯	◯	◯	◯
Personnel Resistance	◯	◯	◯	◯	◯	◯	◯	◯	◯	◯	◯

Please indicate how significant the following factors may be as barriers to providing immunization services (*excluding COVID-19 immunizations*) in your practice setting.

	0 = not a barrier	1	2	3	4	5	6	7	8	9	10 = significant barrier
Vaccine Cost (i.e. purchasing)	◯	◯	◯	◯	◯	◯	◯	◯	◯	◯	◯
Vaccine Storage	◯	◯	◯	◯	◯	◯	◯	◯	◯	◯	◯
Space Availability	◯	◯	◯	◯	◯	◯	◯	◯	◯	◯	◯
Staffing	◯	◯	◯	◯	◯	◯	◯	◯	◯	◯	◯
Personnel Resistance	◯	◯	◯	◯	◯	◯	◯	◯	◯	◯	◯
Reimbursement Rates	◯	◯	◯	◯	◯	◯	◯	◯	◯	◯	◯
Time limitations	◯	◯	◯	◯	◯	◯	◯	◯	◯	◯	◯
Patient Willingness	◯	◯	◯	◯	◯	◯	◯	◯	◯	◯	◯
Practice Regulations	◯	◯	◯	◯	◯	◯	◯	◯	◯	◯	◯

Does your practice setting report immunizations in any way outside of filing for reimbursement? *Check all that apply.*

- ☐ NO - no reporting is done
- ☐ YES - for pediatric patients we report to the state immunization registry
- ☐ YES - for pediatric patients we report to the primary care provider
- ☐ YES - for adult patients we report to the state immunization registry
- ☐ YES - for adult patients we report the immunization to the primary care provider
- ☐ UNCERTAIN - I am uncertain if or how my practice setting reports immunizations
- ☐ Other - please describe:

Did your practice setting change reporting practices related to immunizations in response to COVID-19?

- ◯ YES - we now report all immunizations and we did not before COVID-19
- ◯ YES - we now report COVID-19 immunization, but not other immunizations
- ◯ NO - we have made no changes related to reporting immunizations
- ◯ Other - please describe: __________

Who is administering COVID-19 vaccinations in your pharmacy practice setting? *Check all that apply.*

- ☐ Pharmacists
- ☐ Pharmacy Students
- ☐ Pharmacy Technicians
- ☐ Other - please describe: __________

We welcome your input! Please share any other comments **about COVID-19 vaccinations in the pharmacy practice setting**.

We welcome your input! Please share any other comments about administration of **non-COVID-19 immunizations in the pharmacy practice setting.**

Block 1

Thank you for your interest in this project. You must be over 18 years of age to participate.
